# Catalytic Wiring of Enzymatic Cascades Using ROS‐Flux‐Regulated Biodegradable Borophene

**DOI:** 10.1002/smll.202512858

**Published:** 2026-02-17

**Authors:** Pranay Saha, Shraddha Krishnakumar, Aysenur Yardim, David Skrodzki, Adrienne Griffiths, Teresa Aditya, Dipanjan Pan

**Affiliations:** ^1^ Department of Nuclear Engineering The Pennsylvania State University State College Pennsylvania USA; ^2^ Department of Biomedical Engineering The Pennsylvania State University State College Pennsylvania USA; ^3^ Department of Bioengineering, Institute of Natural Sciences Ege University Izmir Türkiye; ^4^ Department of Materials Science and Engineering The Pennsylvania State University State College Pennsylvania USA; ^5^ Department of Chemistry The Pennsylvania State University State College Pennsylvania USA; ^6^ Huck Institutes of the Life Sciences State College Pennsylvania USA

**Keywords:** borophene, electrochemical biosensing, enzymatic catalysis, glucose oxidase‐horseradish peroxidase (GOx‐HRP) cascade, reactive oxygen species (ROS)

## Abstract

Two‐dimensional (2D) borophene, a single‐atom‐thick allotrope of boron, exhibits exceptional conductivity, anisotropy, and chemical reactivity, yet very little is known about its electrochemical properties. Here, we delineate its enzymatic and electrochemical behavior under biologically relevant redox conditions. Spectroscopic and microscopic studies reveal concentration‐dependent degradation of borophene by hydrogen peroxide (H_2_O_2_), yielding boronic acids, confirmed by a curcumin‐rosocyanine assay. Enzymatic cascades employing glucose oxidase and horseradish peroxidase establish borophene as both an electrocatalyst and a chemically responsive transducer, generating dual colorimetric and electrochemical outputs. Electroanalytical measurements (cyclic voltammetry, chronoamperometry, and differential pulse voltammetry) show that borophene efficiently wires peroxidase reactions by sensitizing H_2_O_2_ detection through borophene–HRP interfaces. In contrast, glucose sensing displays diminished currents due to reactive oxygen species–mediated passivation. These results position borophene as a unique platform for catalytic wiring of enzyme cascades in which ROS flux dynamically regulates signal output, enabling transient, self‐reporting biosensors and motivating stabilization strategies for long‐term bioelectronic integration.

## Graphical abstract

Borophene‐modified electrodes facilitate enhanced electron transfer relative to bare electrodes, enabling efficient electrochemical communication with horseradish peroxidase (HRP) and glucose oxidase (GOx). The borophene–enzyme interface promotes electrocatalytic wiring of redox cascades, yielding amplified current outputs and positioning borophene as a responsive bioelectronic transducer under ROS‐mediated conditions.

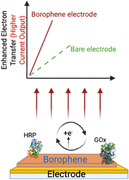

## 1 Introduction

Borophene, a single‐atom‐thick sheet of boron atoms, has emerged as a promising 2D material owing to its remarkable electronic conductivity, mechanical flexibility, and chemical reactivity [1, 2, 3]. Unlike other 2D materials, borophene exhibits polymorphic structures with anisotropic properties, making it highly tunable for a range of applications, including energy storage, catalysis, and biomedical technologies [4, 5, 6, 7, 8, 9]. However, experimental studies evaluating specific key catalytic behaviors, such as reactive oxygen species (ROS) generation and electron transfer mediation, are lacking [10, 11, 12, 13].

Given the pivotal role of enzymatic catalysis in a wide range of bio‐applications, understanding the interaction of borophene with key biological redox reactions is essential [14]. Recent studies related to 2D materials, such as graphene, have shown that enzymatic activity and oxidative stress influence their degradation and catalytic properties [7, 13, 15, 16, 17]. Notably, Myeloperoxidase (MPO), an enzyme released by immune cells, catalyzes oxidative degradation pathways in materials such as graphene. The distinct yet similar physicochemical properties of borophene to graphene suggest that similar enzymatic interactions that undermine the electrocatalytic performance of borophene may occur. Recent studies have identified borophene‐based nanozymes as promising platforms for glucose sensing, combining green synthesis with dual GOx‐ and peroxidase‐like activities to enable sensitive detection in complex media [18, 19, 20]. Complementary colloidal and materials engineering investigations further demonstrate that two‐dimensional boron nanostructures can be stably dispersed in aqueous environments with tunable surface chemistry, while alloying and composite strategies enhance their electronic conductivity and catalytic activity.

**SCHEME 1:**
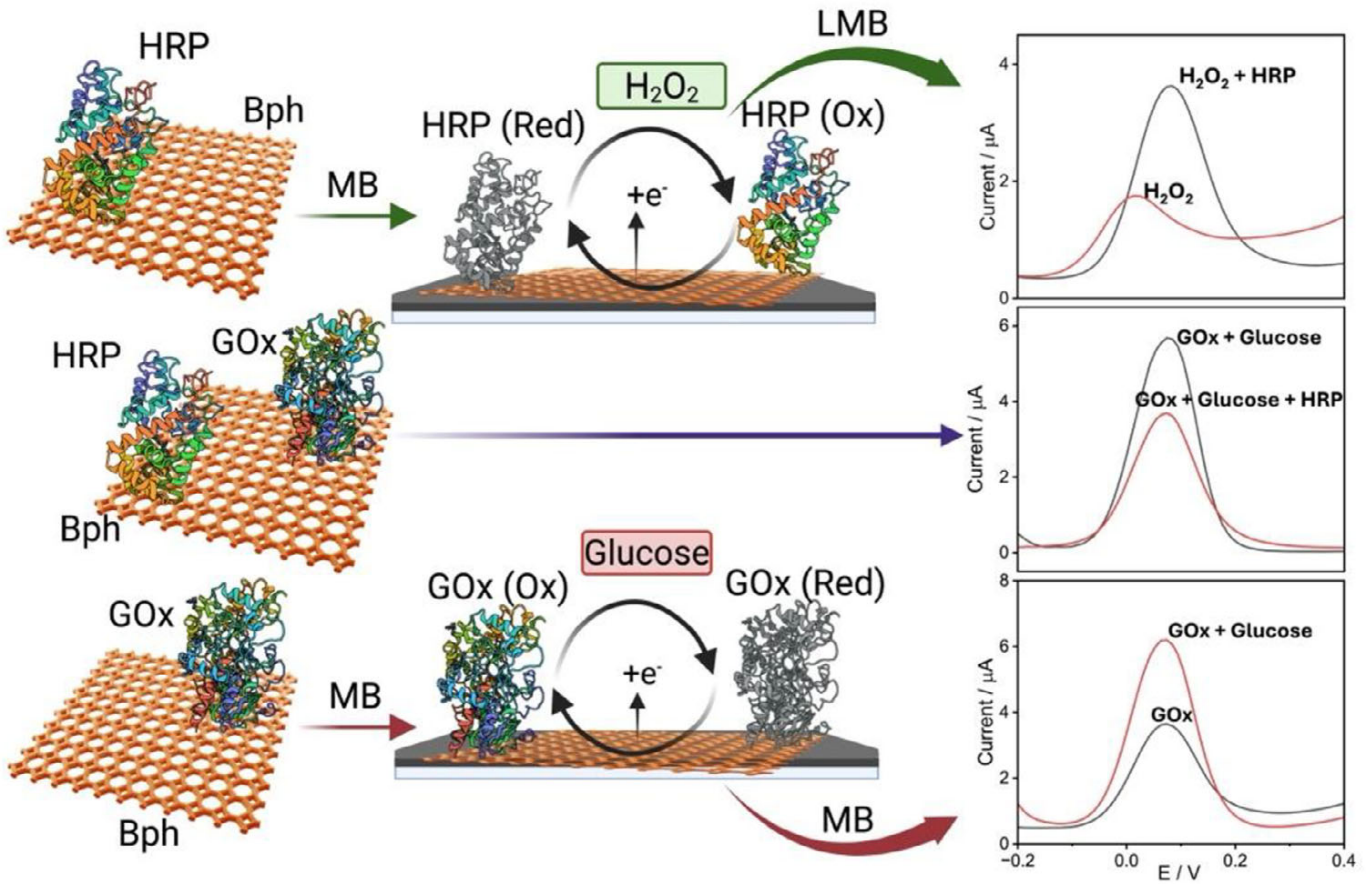
ROS‐controlled catalytic wiring of enzymatic cascades on a borophene‐modified electrode. In the HRP/H_2_O_2_ system, borophene in combination with methylene blue (MB) facilitates efficient electron transfer, thereby enhancing current through leucomethylene blue (LeucoMB) redox cycling. In the GOx/glucose system, enzymatically produced H_2_O_2_ drives a cascade with HRP, while GOx alone yields attenuated signals due to oxidative passivation of borophene. The differential pulse voltammetry (DPV) responses (right) highlight borophene's dual role as a catalytic transducer and ROS‐responsive degradable platform. Key: Bph—Borophene; GOx—Glucose Oxidase; HRP—Horse Radish Peroxidase; MB—Methylene Blue; LeucoMB—Leucomethylene Blue; (Ox)—Oxidized form; (Red)—Reduced form.

Here, we investigate the enzymatic catalytic behavior of borophene using both photophysical analyses and electrochemical studies on electrode surfaces, leveraging its exceptional electron‐transfer capacity for biosensing applications. Specifically, we study its role as a platform for enzymatic biosensors using glucose oxidase (GOx) and horseradish peroxidase (HRP) to detect glucose and hydrogen peroxide (H_2_O_2_), respectively (Scheme 1). Our results indicated that borophene can serve as an effective electrocatalyst in redox environments, particularly for H_2_O_2_ sensing, with the signal originating from the electro‐reduction of the mediator, methylene blue (MB). However, in oxidative conditions, borophene undergoes structural modifications, influencing its catalytic efficiency. For the first time, these findings elucidate the mechanistic basis underlying borophene's stability and functionalization, providing critical design principles for its use in bioelectrochemical applications and enabling the development of highly sensitive biosensors and enzyme‐responsive platforms.

## 2 Results and Discussion

### 2.1 Synthesis and Structural Characterization of Borophene

Borophene in the β12 allotrope was synthesized in our laboratories following a recently published work by our group [21, 22]. Atomic force microscopy (AFM) images (Figure S1a) supported the successful exfoliation of bulk boron powder into borophene nanosheets. The chemical composition of borophene nanosheets was confirmed in scanning electron microscopy‐energy dispersive X‐ray spectroscopy (SEM‐EDS) elemental mapping (Figure S1b). 2D borophene was purified through centrifugation and dialysis for further studies. The structural integrity and oxidative transformation of borophene under ROS exposure were systematically investigated using complementary imaging and spectroscopic physicochemical characterization techniques.

**FIGURE 1:**
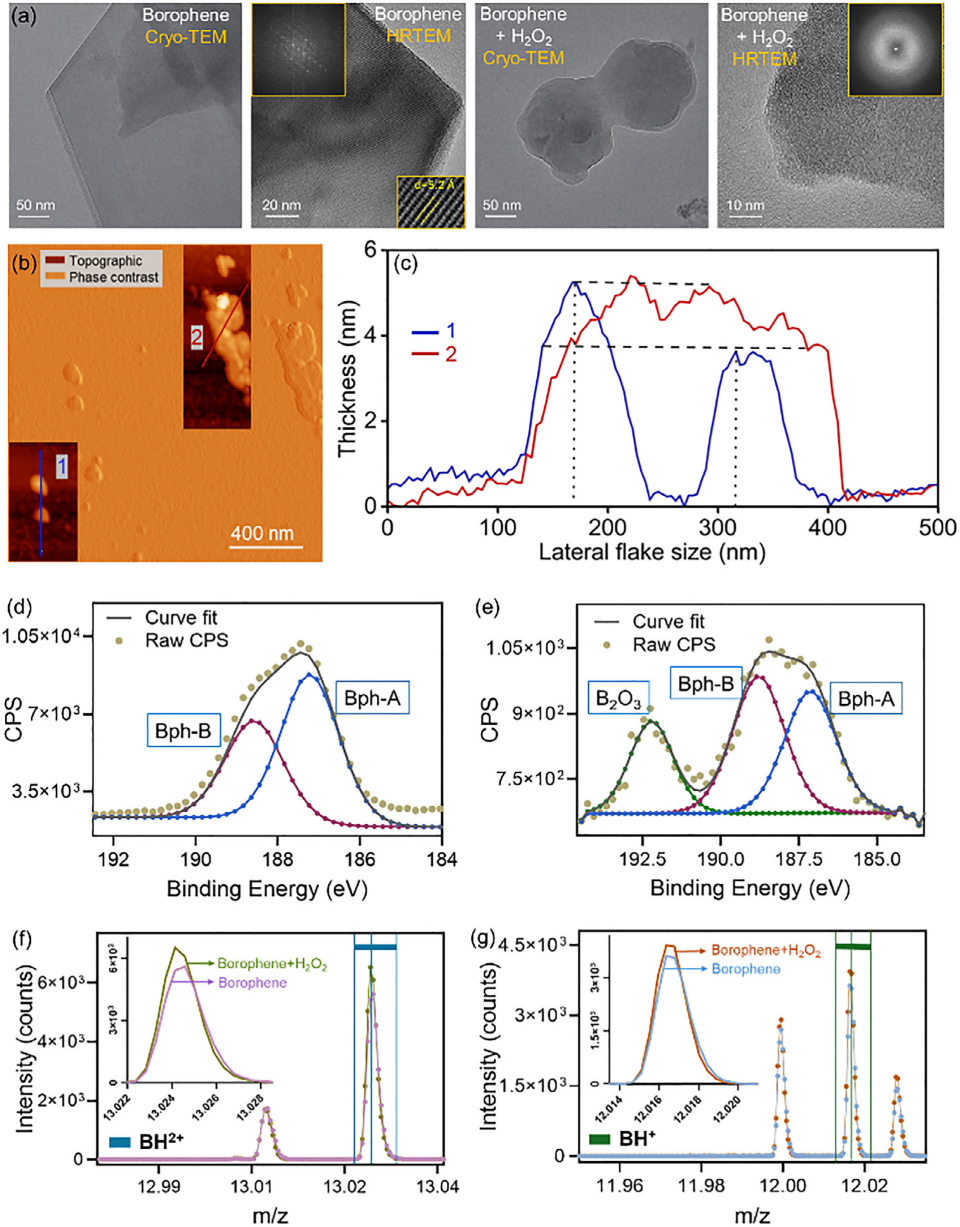
Structural and oxidative transformation of borophene under ROS exposure. (a) Cryo‐transmission electron microscopy (TEM) and high‐resolution TEM images of pristine borophene reveal crystalline lattice fringes (inset diffraction pattern). At the same time, exposure to H_2_O_2_ induces morphological distortion and loss of long‐range order, confirming oxidative degradation. (b) AFM topographic and phase‐contrast images showing lateral dimensions and surface morphology of borophene flakes (insets highlight representative flakes), (c) Corresponding height profiles indicating nanosheet thicknesses of ∼3–5 nm with lateral dimensions in the 100–300 nm range; (d,e) High‐resolution B 1s X‐ray photoelectron spectroscopy spectra of pristine (left) and oxidized (right) borophene, showing the emergence of B_2_O_3_ species and shifts in Bph‐A/Bph‐B components after H_2_O_2_ treatment; (f,g) High‐resolution secondary‐ion mass spectroscopy data demonstrating the generation of boron oxide and boronic acid fragments (BH^2+^ and BH^+^) upon H_2_O_2_‐mediated oxidation, higher than pristine borophene.

UV‐vis spectroscopy showed a concentration‐dependent decrease in borophene absorbance, indicating increased optical transparency with increasing H_2_O_2_ levels (Figure S2a). This trend suggests progressive oxidative conversion of borophene into boronic acid species. Cryo‐TEM analysis of pristine borophene nanosheets revealed well‐defined two‐dimensional flakes with preserved nanosheet‐like morphology (Figure 1a). High‐resolution TEM further confirmed crystalline lattice fringes (0.52 nm), and the corresponding FFT diffraction patterns demonstrated long‐range order characteristic of pristine borophene (β12) domains [23, 24]. AFM topographic and phase‐contrast imaging provided a quantitative assessment of nanosheet dimensions (Figure 1b). The borophene flakes exhibited lateral sizes of ∼100–300 nm with thicknesses of ∼3–5 nm, consistent with multilayer sheet architecture (Figure 1c).

### 2.2 Oxidative Transformation of Borophene Under ROS Exposure

Upon treatment with H_2_O_2_, marked morphological distortion was observed in both TEM and AFM images, with blurred lattice fringes and partial collapse of the sheet structure, signifying oxidative degradation of the borophene framework. The apparent surface roughness and step‐like profiles became more pronounced following H_2_O_2_ exposure, further suggesting ROS‐induced oxidative etching and thinning. In Figure 1c, degradation of stacked multilayer borophene into smaller nanosheets is evident. Chemical fingerprints of this transformation were captured by high‐resolution XPS of the B1s region (Figure 1d,e). In pristine borophene, Bph‐A and Bph‐B peaks corresponding to distinct boron bonding states dominated the spectra. Upon oxidation, these components decreased in intensity with the concurrent emergence of a strong B_2_O_3_ feature, consistent with the conversion of elemental boron states into oxidized boron oxide species. This finding highlights the borophene lattice's susceptibility to ROS‐mediated bond cleavage and surface oxidation.

SIMS analysis corroborated these results (Figure 1f,g). Pristine borophene exhibited low intensities of fragmented boron species, whereas ROS‐treated samples showed distinct signatures of boron oxide and boronic acid fragments (BH^2+^ and BH^+^). The increased abundance of oxidative fragments provides direct evidence of borophene's chemical transformation into oxidized derivatives under H_2_O_2_‐induced stress. Collectively, the multimodal datasets demonstrate that borophene undergoes rapid morphological deformation, progressive loss of crystallinity, and sequential chemical oxidation upon exposure to reactive oxygen species, yielding boron oxides and boronic acids that define its degradation pathways and govern its nano‐bio interfacial reactivity. The observed trends align with 2D materials’ reactivity toward oxidative agents, in which surface defects and edge sites serve as primary reaction centers for oxidation [2, 25, 26].

### 2.3 ROS‐Mediated Degradation Pathway and Environmental Stability

Borophene's unique anisotropic structure and electron‐rich surface make it highly susceptible to oxidative degradation, particularly in aqueous environments containing reactive oxygen species (ROS) such as H_2_O_2_. This suggests that borophene's stability in oxidative environments is critically dependent on its concentration and surface chemistry [27]. Oxidation proceeds aggressively at lower concentrations of borophene or other 2D materials, supposedly due to higher relative exposure to ROS [28, 29, 30]. The surface oxidation properties of 2D materials are particularly relevant for potential applications of borophene in biosensing, catalysis, and energy storage, where stability under oxidative conditions is crucial [31, 32, 33]. Like other 2D materials, the oxidation‐induced structural breakdown of borophene limits its functional lifespan, making it an excellent choice for degradable materials in bio‐applications, for example, implantable.

Borophene remains stable in aqueous media for 30 min to several hours, depending on the level of exfoliation, concentration, surface chemistry, etc., [34, 35]. Though borophene undergoes progressive oxidation to boronic acid species with increasing H_2_O_2_ concentrations. This observation is similar to phosphorene oxidative pathways, in which surface‐exposed phosphorus atoms react with ROS, leading to hydrolysis and the formation of phosphoric acid and phosphorus oxides [36, 37]. The structural transformations observed in reaction vials further support this hypothesis, with initially dispersed borophene suspensions exhibiting aggregation, sedimentation, and color fading, indicating altered electronic properties and structural integrity (Figure S1).

The degradation likely begins with H_2_O_2_‐derived ROS disrupting borophene's planar structure, forming hydroxyl and peroxide groups that hydrolyze into boronic acids [38]. This process critically affects borophene's stability and reactivity in oxidative environments, especially in biological and catalytic settings.

### 2.4 Quantification of Boronic Acid Formation During Borophene Degradation

**FIGURE 2:**
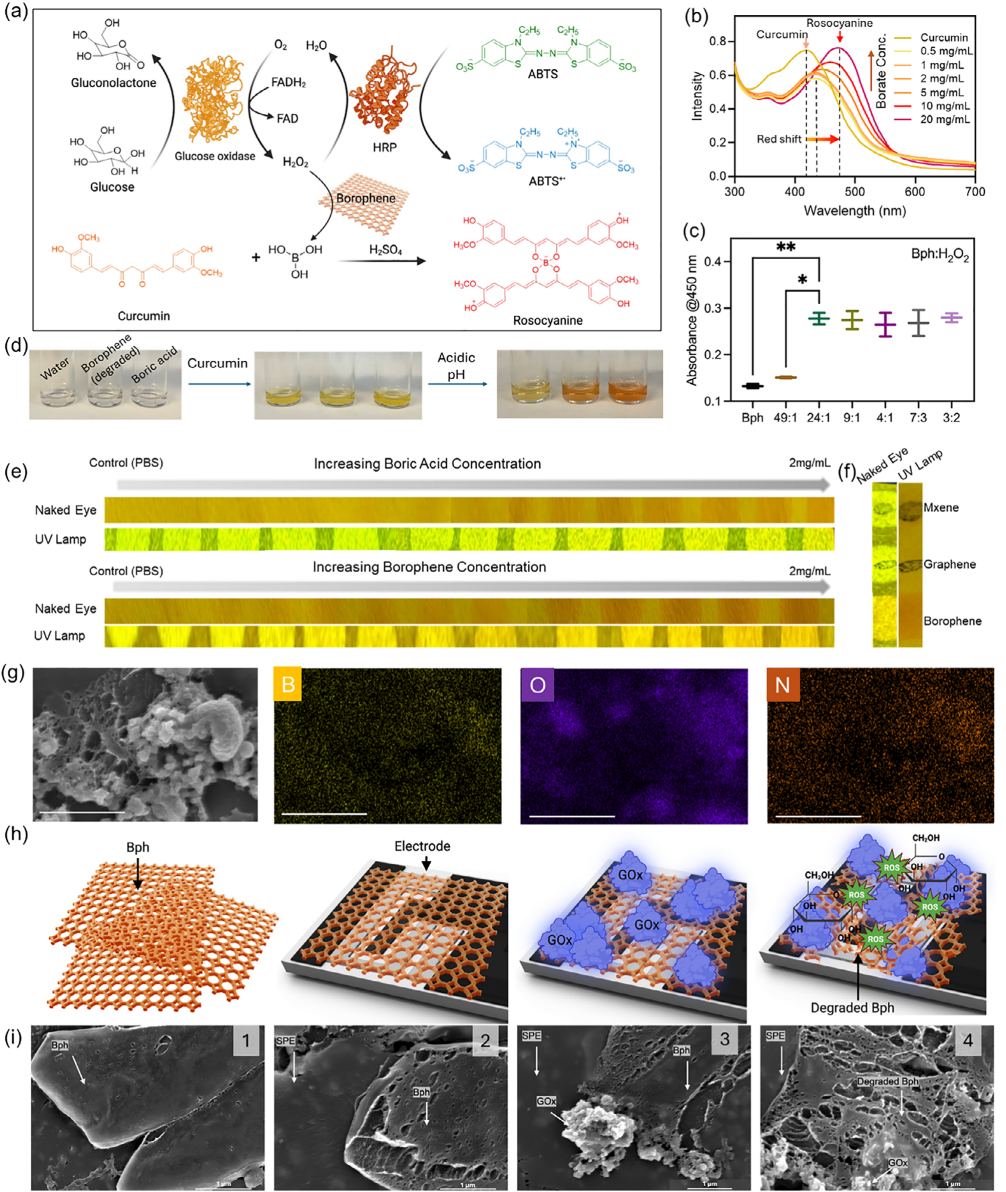
Enzymatic cascade‐driven oxidative degradation of borophene. (a) Schematic of glucose oxidase (GOx)‐horseradish peroxidase (HRP) cascade in the presence of borophene, where enzymatically generated H_2_O_2_ drives borophene oxidation and simultaneous signal generation via ABTS oxidation (blue) and curcumin‐boronic acid complexation to form rosocyanine (red). (b) Spectroscopic assay of concentration‐dependent borate‐curcumin complex formation. (c) Absorbance profiles confirming quantitative formation of rosocyanine in the presence of degraded borophene with significance marked as *p < 0.05; **p < 0.01 assessed through ANOVA test. (d) Change in color of curcumin with degraded borophene in acidic pH (e) Self‐reporting optical response of borophene degradation using a curcumin‐infused paper strip, and the results are compared with boric acid standards. (f) Visual response highlights borophene‐specific behavior compared to graphene and MXene. (g) SEM‐EDX elemental mapping of GOx on borophene‐coated electrodes exhibits B, O, and N distributions, confirming protein immobilization. (h) Schematic representation of borophene‐modified carbon screen‐printed electrodes (CSPEs) and subsequent enzyme loading and ROS‐mediated borophene degradation. (i) SEM images of (1) borophene nanosheets, (2) borophene over screen‐printed electrodes (SPE), (3) GOx on borophene‐SPE, (4) GOx‐glucose mediated (ROS) degradation of borophene.

Boronic acids formed during borophene degradation under ROS were quantified using curcumin as a selective probe, enabling the development of a quantitative assay that correlates this degradation with subsequent electrochemical nanozymatic cascade activity. To evaluate this chemistry under biologically relevant enzymatic conditions, the curcumin–rosocyanine and ABTS assays were extended to a glucose oxidase (GOx)‐ glucose‐HRP‐driven system without exogenous H_2_O_2_ (Figure 2a). Formation of the rosocyanine complex, marked by a bathochromic absorbance shift from ∼420 to ∼470 nm, directly indicates the concentration of boronic acids released during borophene degradation. (Figure 2b,c). At low boronic acid levels, the complex exhibited an absorption maximum at ∼435 nm, whereas higher concentrations resulted in a red shift toward 470 nm, consistent with increased rosocyanine formation. Absorbance spectra across various borophene: H_2_O_2_ ratios confirmed substantial oxidative degradation, with liberated boronic acids reacting with curcumin in the presence of H_2_SO_4_ to yield the rosocyanine complex. Among the tested conditions, a borophene:H_2_O_2_ ratio of 24:1 produced the most pronounced shift to 470 nm, indicating maximal boronic acid generation (Figure 2c). Figure 2d exhibits a visible change in color for the curcumin to borate‐curcumin (rosocyanine) complex formation at acidic pH.

GOx oxidized glucose to generate H_2_O_2_, which both oxidized borophene to boronic acids, forming a red rosocyanine complex with curcumin, and served as a substrate for HRP to oxidize ABTS into its blue‐green radical cation. The integrated GOx‐HRP cascade thus produced dual colorimetric signals. (Figure 2a–d, Figure S2b,c). For easier optical monitoring, curcumin‐infused paper (reporter dye) was used, which exhibited a clear, concentration‐dependent color change with increasing boric acid concentration under both visible and UV light, indicating a robust optical readout suitable for semi‐quantitative analysis. Under identical conditions, borophene produced a pronounced and consistent color signature (Figure 2e) that matches this calibration trend, supporting a degradation‐driven “self‐reporting” mechanism where borophene oxidation generates boric acid/borate species that trigger the curcumin color transition. In contrast, MXene and graphene at equivalent concentrations showed negligible or qualitatively different changes, highlighting that the optical response is specific to borophene rather than a generic effect of 2D material deposition (Figure 2f).

We benchmarked borophene‐derived samples against aqueous boric acid standards using HPLC. The resulting chromatographic profiles exhibit coincident boric‐acid‐associated features (Figure S2d–f) (i.e., matching retention behavior) in both the boric acid control (Figure S2d) and the borophene sample (Figure S2e,f). This agreement supports the presence of boric acid as a degradation product and is consistent with oxidative/hydrolytic conversion of borophene under water and H_2_O_2_ exposure.

**FIGURE 3:**
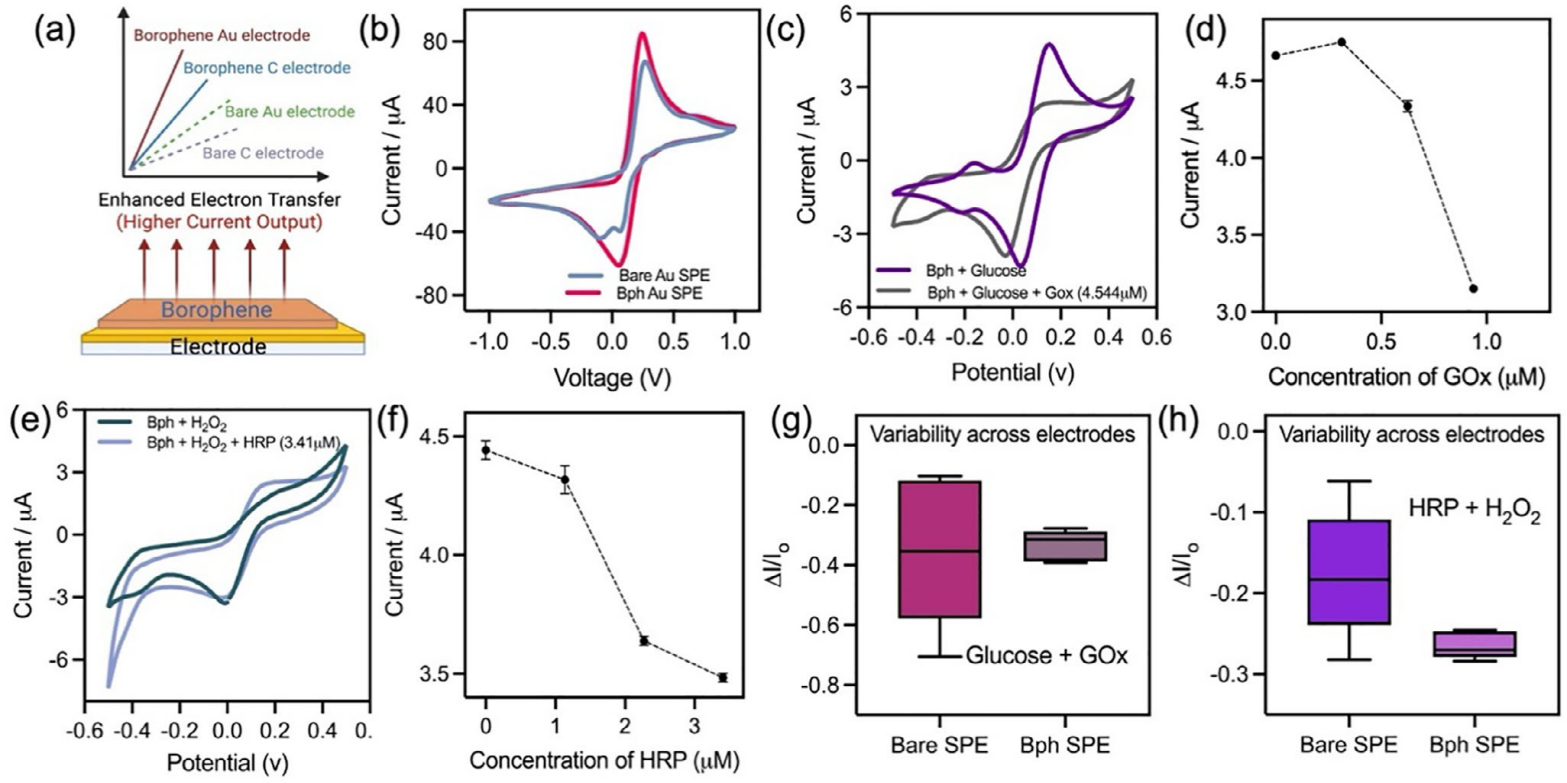
Electrochemical performance of borophene‐modified electrodes with ROS generation and neutralization. (a) Schematic of enhanced electron transfer on borophene‐modified Au and carbon electrodes compared to bare electrodes. (b) CV showing improved conductivity of borophene‐Au SPE versus bare Au. (c,d) GOx–glucose system showing signal attenuation with oxidative passivation and concentration dependence. (e,f) HRP‐H_2_O_2_ system displaying enzyme‐enhanced catalytic reduction with concentration‐dependent current response. (g, h) Variability plots demonstrating higher sensitivity and reproducibility of borophene‐modified electrodes compared to bare SPEs (n = 5) and results are expressed as mean ± SD.

**FIGURE 4:**
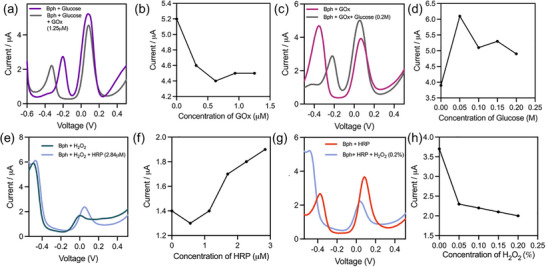
Electrochemical characterization of borophene‐mediated enzymatic cascades under ROS‐regulated conditions. (a,b) Differential pulse voltammetry (DPV) and concentration‐dependent current plots for GOx‐glucose on borophene, showing progressive current attenuation with increasing GOx due to ROS‐driven oxidative passivation (mean, n = 5). (c,d) DPV and calibration of HRP–H_2_O_2_ systems, demonstrating concentration‐dependent current enhancement and efficient electron transfer at borophene‐coated electrodes (mean, n = 5). (e,f) Electrochemical response to varying glucose concentrations in the Gox‐borophene system, highlighting reduced current output at higher glucose levels, consistent with increased ROS flux (mean, n = 5). (g,h) Borophene‐HRP responses with varying H_2_O_2_ concentrations, confirming peroxide‐driven modulation of catalytic currents (mean, n = 5).

Morphological and surface analyses confirmed the interplay between enzyme loading and borophene degradation. SEM‐EDS elemental mapping of borophene‐modified electrodes revealed uniform distributions of boron, oxygen, and nitrogen, consistent with successful GOx immobilization (Figure 2g). Schematic and SEM imaging further illustrate the progression from pristine borophene nanosheets (Figures 2h,i‐1) to their integration onto screen‐printed electrodes (SPEs, Figure 2i‐2), followed by enzyme adsorption (Figure 2i‐3), and finally, ROS‐mediated structural degradation under glucose stimulation (Figure 2i‐4).

### 2.5 Electrochemical Characterization of Borophene‐Modified Electrodes

To elucidate the role of borophene as a catalytic transducer in enzymatic cascades, electrochemical measurements were carried out using K_3_[Fe(CN)_6_]/K_4_[Fe(CN)_6_] (2.5 mM each) as the redox couple and methylene blue (MB, 100 µM) as the redox mediator [39, 40, 41, 42]. These dual mediators enable sensitive monitoring of electron transfer across enzyme–electrode interfaces. The electrochemical response of borophene‐coated electrodes was systematically compared to bare electrodes in the presence of glucose oxidase (GOx)/glucose and horseradish peroxidase (HRP)/H_2_O_2_ systems to probe how ROS flux regulates borophene's catalytic performance. Cyclic voltammetry (CV) measurements confirmed that borophene integration onto both gold and carbon screen‐printed electrodes (SPEs) markedly enhanced electron transfer relative to the bare electrodes, as illustrated by the higher current outputs and sharper redox features (Figure 3a,b, Figure S3a,b). The enhanced conductivity underscores borophene's effectiveness as a transduction layer. Additional measurements were performed in saliva, a more complex matrix containing diverse ionic and organic components, to check the feasibility of the platform in biological fluids. The platform preserves its characteristic electrochemical response in this medium (Figure S3c,d), with well‐resolved current‐potential features comparable to those observed in PBS. CV measurements over bare SPEs are shown in Figure S4a–d.

In the GOx‐glucose system, however, progressive attenuation of current density was observed at borophene‐modified electrodes as enzyme concentration increased (Figure 3c,d). This “signal‐off” response can be attributed to oxidative degradation of the borophene surface by enzymatically generated H_2_O_2_, which degrades the conductive lattice into boronic acid species and disrupts electron transport pathways. This effect demonstrates borophene's intrinsic ROS‐responsiveness and its susceptibility to enzymatic oxidative environments.

In contrast, the HRP‐H_2_O_2_ system exhibited catalytic current enhancement with increasing HRP concentrations, consistent with enzyme‐facilitated reduction of H_2_O_2_ (Figure 3e,f). The contrasting current attenuation in the GOx cascade and amplification in the HRP system highlights borophene's dual electrochemical roles: acting as a degradable transducer in ROS‐generating reactions and as a catalytic mediator in ROS‐consuming reactions. Further variability analysis revealed that borophene‐modified electrodes exhibited higher reproducibility and sensitivity than bare SPEs in both the GOx‐glucose and HRP‐H_2_O_2_ systems (Figure 3g,h). This reproducibility, together with borophene's ROS‐flux–dependent reactivity, defines it as a distinctive electrochemical interface that integrates degradability with signal transduction.

### 2.6 ROS‐Flux‐Regulated Enzymatic Cascades and Catalytic Wiring

**FIGURE 5:**
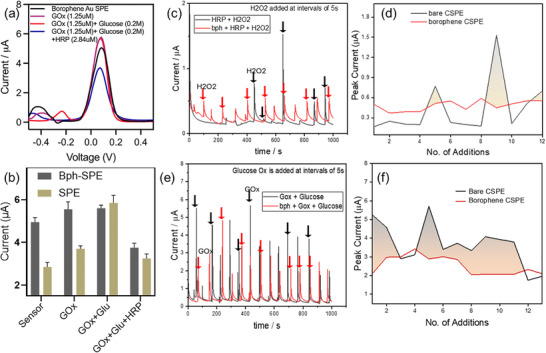
(a) Comparative voltammograms of sequential enzyme cascades (GOx ± glucose ± HRP) on borophene‐modified Au SPEs, revealing stepwise changes in current reflecting cascade complexity. (b) Histogram comparing current responses for GOx–glucose systems on bare and borophene‐modified electrodes, showing signal attenuation due to ROS‐driven oxidative passivation (n = 5) andresults are expressed as mean ± SD. (c–f) Chronoamperometric traces and statistical variability analyses across electrodes for Gox‐glucose and HRP‐H_2_O_2_ systems, showing higher reproducibility and sensitivity for borophene‐modified electrodes compared to bare controls.

DPV resolved borophene‐mediated enzyme wiring under ROS control. In GOx–glucose‐HRP experiments (Figure 4a–d, Figure S5a–d), higher GOx or glucose attenuated current, consistent with ROS‐driven oxidative passivation and reduced electron transfer. In the HRP–H_2_O_2_ system (Figure 4e–h), H_2_O_2_ titrations at borophene electrodes produced proportional catalytic currents; omission of HRP or H_2_O_2_ gave negligible signals, attributing responses to enzymatic turnover. With H_2_O_2_ fixed, increasing HRP boosted current without shifting peak potential, then plateaued at high loadings—signaling a shift from enzyme‐limited to diffusion/film‐limited kinetics. Sequential cascades (Figure 5a) showed stepwise current changes as components were added, reflecting rising ROS flux. Borophene outperformed bare electrodes (Figure 5b) but was more prone to attenuation under sustained ROS. Overall, borophene's output tracks enzyme efficiency and ROS burden, highlighting a trade‐off between signal gain and interface stability in 2D‐material biosensors.

Time‐resolved amperometry (Figure 5c–f) exposes a dual response of borophene. In HRP‐H_2_O_2_, sequential peroxide additions at borophene electrodes yield larger, cleaner current steps with faster rise/decay rates than on bare carbon, indicating more efficient interfacial electron transfer and catalytic turnover. In the GOx‐glucose cascade, by contrast, currents on borophene attenuate relative to bare controls, consistent with in situ H_2_O_2_ generation that oxidizes the borophene film and depresses its conductivity. Variability analyses (Figure 5d,f) show a narrower inter‐electrode spread for borophene, indicating superior device‐to‐device reproducibility. Moreover, the uniform distribution of GOx on the borophene surface ensures stable, localized enzymatic activity, resulting in consistent chronoamperometric signals over time. In contrast, free GOx in solution exhibits variable diffusion and interaction with the electrode, resulting in less reproducible current responses. Overall, borophene markedly enhances direct peroxide sensing yet is vulnerable under sustained ROS, highlighting a sensitivity‐durability trade‐off that should guide future 2D‐electrode design.

Borophene was further compared with graphene and MXene‐modified electrodes fabricated using the same protocol and evaluated under identical GOx‐glucose catalytic conditions. As shown in Figure S6, graphene and MXene exhibited gradual and stable amperometric responses, consistent with their relatively inert electrochemical behavior, where signal evolution was dominated by surface adsorption and charge transport. In contrast, borophene displayed a rapid and markedly higher current response upon glucose addition. This behavior originated from its highly electroactive and chemically interactive surface, which promoted efficient charge transfer while undergoing progressive oxidation during the GOx‐glucose‐H_2_O_2_ cascade. The in situ‐generated H_2_O_2_ partially oxidized borophene to boric/borate species, transiently enhancing electron transfer before gradual degradation reduces the effective electroactive surface. Consequently, borophene exhibited the highest peak current and relative current change (ΔI/I_0_), followed by graphene and MXene, indicating that sensor performance was governed by both enzyme‐electrode coupling and the electrocatalytic reactivity of the support under continuous ROS generation.

Additional comparative data is provided in Figures S7 and S8. Borophene‐modified electrodes showed slightly improved stability and reproducibility over 45 min compared to graphene and MXene (Figure S7). Differential pulse voltammetry under enzymatic cascade conditions further confirmed higher current output and larger signal modulation for borophene (Figure S8). Limits of detection and quantification (LOD and LOQ) were extracted using linear and nonlinear fitting, as shown in Figure S8e, exhibiting better LOD and LOQ values for borophene‐modified electrodes.

### 2.7 Mechanistic Implications and Design Principles

Taken together, these electroanalytical studies establish borophene as a unique ROS‐flux–regulated platform for catalytic wiring of enzymatic cascades. In the HRP‐H_2_O_2_ system, borophene acts as an efficient electron‐transfer scaffold, enabling sensitive and reproducible peroxide detection. Conversely, in the GOx–glucose cascade, enzymatically produced ROS induce oxidative passivation of borophene, attenuating signals but simultaneously providing a self‐reporting degradation pathway. This dual behavior‐signal amplification under direct peroxide flux and suppression under enzymatic ROS accumulation demonstrates borophene's potential as both a catalytic enhancer and a biodegradable, enzyme‐responsive transducer (Scheme 1). Furthermore, these findings establish a mechanistic framework for deploying borophene in dynamic bio‐electrochemical platforms where ROS flux governs sensitivity, stability, and functional lifespan. Reaction schemes were developed to connect the underlying chemistry with the observed charge‐transfer behavior, based on enzyme‐cascade–mediated borophene degradation kinetics and supported by photophysical, electrochemical, SIMS, and HPLC‐MS results. In the GOx/FAD‐catalyzed oxidation of glucose, electron consumption leads to a decrease in current, which can be more pronounced on borophene‐modified electrodes due to additional electron withdrawal at the interface. As the reaction proceeds, however, the generation of electroactive products and improved interfacial electron transport can produce a net signal increase. In this regime, borophene enhances the response by offering a high‐surface‐area interface with strong affinity for redox species, enriching their local concentration and facilitating more efficient interfacial charge transfer compared with a bare electrode under identical sensing conditions.

**Table:** 

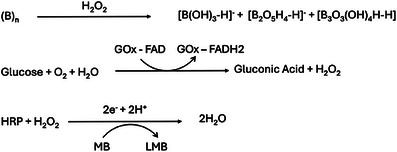

## 3 Conclusion

In conclusion, we show that borophene operates as a bidirectional platform in enzymatic redox environments, serving not only as an efficient electron‐transfer interface for wiring catalytic cascades but also as a chemically responsive transducer where reactivity is dictated by ROS flux. Electrochemical studies revealed that borophene enhances H_2_O_2_ detection through HRP‐mediated reduction while undergoing oxidative transformation into boronic acid derivatives, which attenuates current responses during glucose sensing. This ROS‐mediated degradation encodes self‐reporting signals, positioning borophene as a biodegradable material uniquely capable of coupling catalytic activity with structural transience. The interplay between conductivity, enzymatic electron transfer, and oxidative instability underscores both the opportunity to exploit borophene for transient, enzyme‐responsive biosensors and the necessity for stabilization strategies to extend operational lifetimes. By unveiling a mechanistic framework for ROS‐regulated catalytic wiring, this study establishes borophene as a previously unrecognized class of 2D material that unites enzymatic catalysis with redox‐active materials chemistry, opening new paradigms for dynamic biosensing, biocompatible nanodevices, and adaptive catalytic systems.

## 4 Experimental Section

### 4.1 X‐Ray Photoelectron Spectroscopy

The samples were drop‐casted multiple times and vacuum‐dried each time to form a thick layer. XPS experiments were performed using a Physical Electronics VersaProbe III instrument equipped with a monochromatic Al kα X‐ray source (hν = 1486.6 eV) and a concentric hemispherical analyzer. Charge neutralization was performed using both low‐energy electrons (<5 eV) and argon ions. The binding energy axis was calibrated using sputter‐cleaned Cu (Cu 2p3/2 = 932.62 eV, Cu 3p3/2 = 75.1 eV) and Au foils (Au 4f7/2 = 83.96 eV). Peaks were charge referenced to the CH*x* band in the carbon 1s spectra at 284.8 eV. Measurements were made at a takeoff angle of 45° with respect to the sample surface plane. This resulted in a typical sampling depth of 3–6 nm (95% of the signal originated from this depth or shallower). Quantification was done using instrumental relative sensitivity factors (RSFs) that account for the X‐ray cross section and inelastic mean free path of the electrons. The analysis size was ∼200 µm in diameter. The analysis was performed in CasaXPS software.

### 4.2 UV‐vis Spectroscopy

The absorbance spectra for the assay with 96‐well plates were recorded on a Biotek Synergy Neo2Microplate Reader, both for endpoint, kinetic, and spectral analyses. Each of the experiments was repeated at least three times, and the average of these spectra was presented. To standardize the assays, two of the samples from each category were selected randomly. The absorbance spectra were then normalized. The highest absorbance value was chosen for each spectrum, and then we divided the absorbance values by that number. The normalized data was then compared to standardize the assay parameters, regardless of the details of the experiment.

### 4.3 Transmission Electron Microscopy

A 7 µL solution of the borophene was added on top of a carbon‐coated copper grid (400 mesh). This was allowed to stay for ≈10 min before being removed with a filter paper and imaged under a transmission electron microscope (FEI Tecnai T12). The tungsten filament with 80 kV accelerating voltage was used for the investigations.

### 4.4 Cryo‐EM Sample Preparation and Data Collection

Carbon‐coated holey carbon grids (Quantifoil R1.2/1.3, 400 mesh; Quantifoil GmbH) were glow‐discharged using a PELCO easiGlow system (Ted Pella, Inc.) operated at 15 mA for 45 s. For vitrification, a Vitrobot Mark IV (Thermo Fisher Scientific) was equilibrated to 4°C and 100% relative humidity. Aliquots of 3.5 µL of sample were applied to freshly glow‐discharged grids, blotted for 3 s, and plunge‐frozen in liquid ethane. Vitrified specimens were imaged on a Talos Arctica transmission electron microscope (Thermo Fisher Scientific) operated at 200 keV with a total electron exposure of 25 e^−^ Å^−^^2^. Micrographs were recorded at nominal magnifications of 73 000× and 36 000×. All cryo‐EM experiments were performed at the Cryo‐EM Core Facility, Huck Institutes of the Life Sciences, Pennsylvania State University (RRID: SCR_024456).

### 4.5 Atomic Force Microscopy

The peak force tapping scans were performed on a Dimension Icon AFM (Bruker) with ScanAsyst Air (Bruker) probes with a nominal tip radius of 2 nm and a spring constant of 0.4 N m^−1^. We performed 20 and 1 µm scans with a probe vibration frequency of 2 kHz, a peak force amplitude of 150 nm, 512 samples per line, and a scan rate of 0.7 Hz. Images and roughness were analyzed with NanoScope Analysis v.3.

### 4.6 Scanning Electron Microscopy

Borophene is drop‐casted on the working electrode surface of the C‐SPE, dried, and then stuck on SEM stubs using double‐sided carbon tape, air‐dried, and then sputter‐coated with iridium to achieve conductivity. For some electrodes, glucose and glucose oxidase were added onto the borophene. The Verios G4 SEM was then used to monitor the surface topography of each of the samples. The field‐emission scanning electron microscopic images were recorded. The respective EDS spectra were also recorded for each of the samples.

### 4.7 Electrochemical Measurements

Electrochemical experiments were performed using a PalmSens potentiostat (PalmSens BV, The Netherlands) equipped with a standard three‐electrode configuration integrated into screen‐printed electrodes (SPEs). Commercial carbon (C‐SPE) and gold (Au‐SPE) screen‐printed electrodes (Zimmer & Peacock, UK) were employed as the working electrodes, with an Ag/AgCl pseudo‐reference and carbon counter electrode embedded in the same platform. Before use, the working surfaces were rinsed with Milli‐Q water and dried under a nitrogen stream. Borophene suspensions were drop‐cast (10 µL) onto the working electrode area and air‐dried to obtain borophene‐modified electrodes (Bph‐SPE). The redox couple potassium ferricyanide/potassium ferrocyanide (K_3_[Fe(CN)_6_]/K_4_[Fe(CN)_6_], 2.5 mM each) and methylene blue (MB, 100 µM) were used as electrochemical mediators in 0.1 M phosphate‐buffered saline (PBS, pH 7.4). Enzymatic assays employed glucose oxidase (GOx, 1.25 µM), horseradish peroxidase (HRP, 2.84 µM), glucose (0.2 M), and H_2_O_2_ (0.2%) as substrates.

Cyclic voltammetry (CV) was conducted in the range of –1.0 to +1.0 V at a scan rate of 50 mV s^−^^1^. Differential pulse voltammetry (DPV) was carried out with a modulation amplitude of 50 mV and a step potential of 4 mV. Chronoamperometry (CA) was performed by successive substrate additions at fixed potentials (0.0 to –0.3 V). All measurements were conducted at room temperature (∼25°C) with at least three independently prepared electrodes to confirm reproducibility.

### 4.8 Statistical Analysis

All photophysical and electrochemical results were obtained from n = 3 and n = 5 independent experiments, respectively, and presented in the form of “mean ± standard deviation (SD)”The GraphPad Prism 10.4.2 (GraphPad Software, USA) and Origin 2024 (OriginLab Corporation, USA) were used for data visualization, variance analysis, one‐way ANOVA, and Tukey's test, reflecting the differences in the data. *, **, ***, **** represented *p* < 0.05, *p* < 0.01, *p*< 0.001, and *p* < 0.0001, respectively. *p* < 0.05 denoted significant, while n.a. and n.s. were regarded as not applicable and not significant.

## Author Contributions

D.P. and P.S. conceived the idea, and D.P., P.S., and S.K. designed all the experiments. P.S. and S.K. performed all the experiments. P.S. and S.K. equally contributed to this work. A.Y. and A.G. helped with the experiments. D.S. performed TEM. T.A. and A.G. provided samples. D.P., P.S., and S.K. analyzed all data and wrote the manuscript. All authors approved the manuscript.

## Conflicts of Interest

Prof. Pan is the founder of three University‐based start‐ups. None of these entities, however, supported this work.

## Supporting information




**Supporting File**: smll72845‐sup‐0001‐SuppMat.docx.

## Data Availability

The data that support the findings of this study are available in the supplementary material of this article.
