# Supplementary material for: Catalytic Wiring of Enzymatic Cascades Using ROS‐Flux‐Regulated Biodegradable Borophene
Source: Small. 2026 Feb 17;22(22):e12858. doi: 10.1002/smll.202512858 (PMC13089092; doi:10.1002/smll.202512858)
Supplement: Supplementary file 1 — Supporting File: smll72845‐sup‐0001‐SuppMat.docx. [file SMLL-22-e12858-s001.docx]

**Supporting Information**

Catalytic Wiring of Enzymatic Cascades Using ROS-flux Regulated Biodegradable Borophene

*Pranay Saha^a^,^†^ Shraddha Krishnakumar^a^,^†^ Aysenur Yardim ^a,f‡^, David Skrodzki^c^, Adrienne Griffiths^b^, Teresa Aditya^a^ & Dipanjan Pan ^a,b,c,d,e^* ^*^

^a^Department of Nuclear Engineering, The Pennsylvania State University, University Park, PA 16802, USA

^b^Department of Biomedical Engineering, The Pennsylvania State University, University Park, PA 16802, USA

^c^Department of Materials Science and Engineering, The Pennsylvania State University, University Park, PA 16802, USA

^d^Department of Chemistry, The Pennsylvania State University, University Park, PA 16802, USA

^e^Huck Institutes of the Life Sciences, 101 Huck Life Sciences Building, University Park, PA 16802, USA

^f^ Department of Bioengineering, Institute of Natural Sciences, Ege University, Izmir 35100, Türkiye

*^†, ‡^* Authors contributed equally


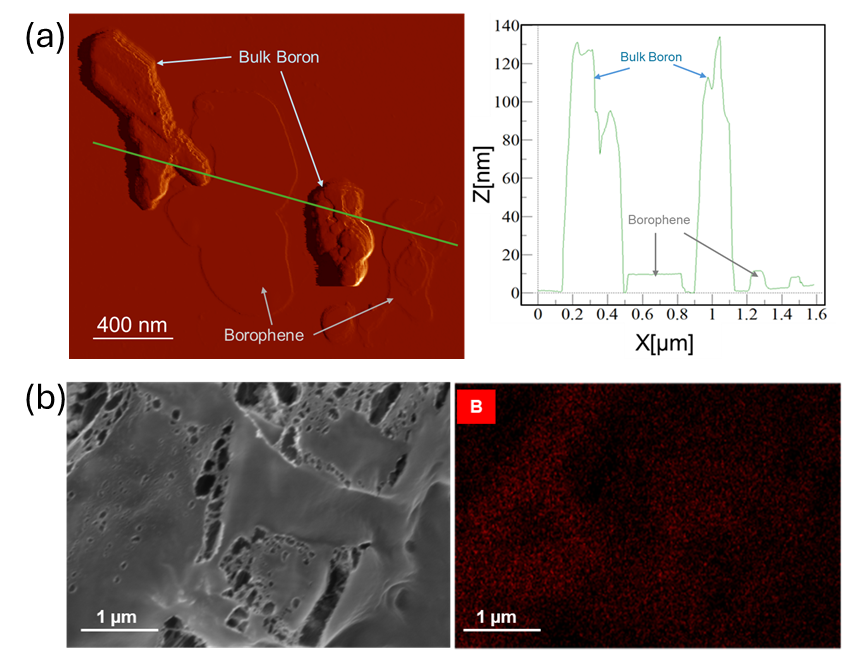


**Figure S1.** (a) AFM images of exfoliated borophene sheets and bulk boron before purification and height profile of both species; (b) SEM-EDS elemental mapping confirms boron in 2D borophene


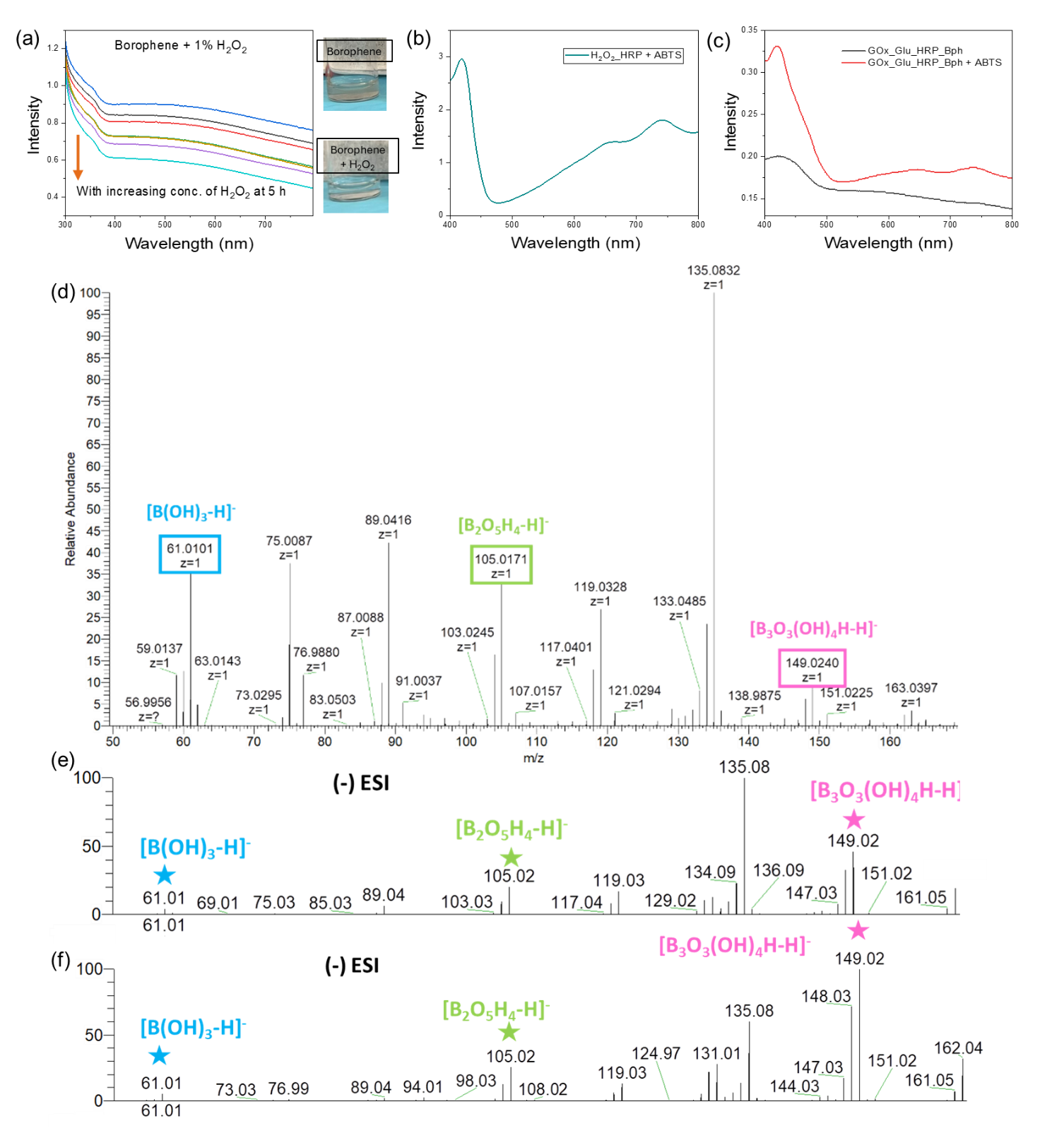


**Figure S2.** (a) Decrease in absorbance intensity of pristine borophene with increasing concentrations of H_2_O_2_ (b) Absorbance of ABTS in during neutralization of H_2_O_2_ in presence of HRP shows generation of blue/teal ABTS•+ (c) Absorbance of ABTS in HRP mediated neutralization of H2O2 produced by glucose oxidase and glucose while in the presence of borophene in media exhibits similar spectrometric trends to monitor generation and neutralization of H_2_O_2_ (d) HPLC-MS for boric acid standards. HPLC-MS factions collected at (e) 5 minutes and (f) 45 minutes incubation in water with catalytic amount of H2O2. Both HPLC fractions confirm aerial/aqueous and hydrogen peroxide mediated oxidation of borophene and its consequent degradation into boric/boronic acid species.


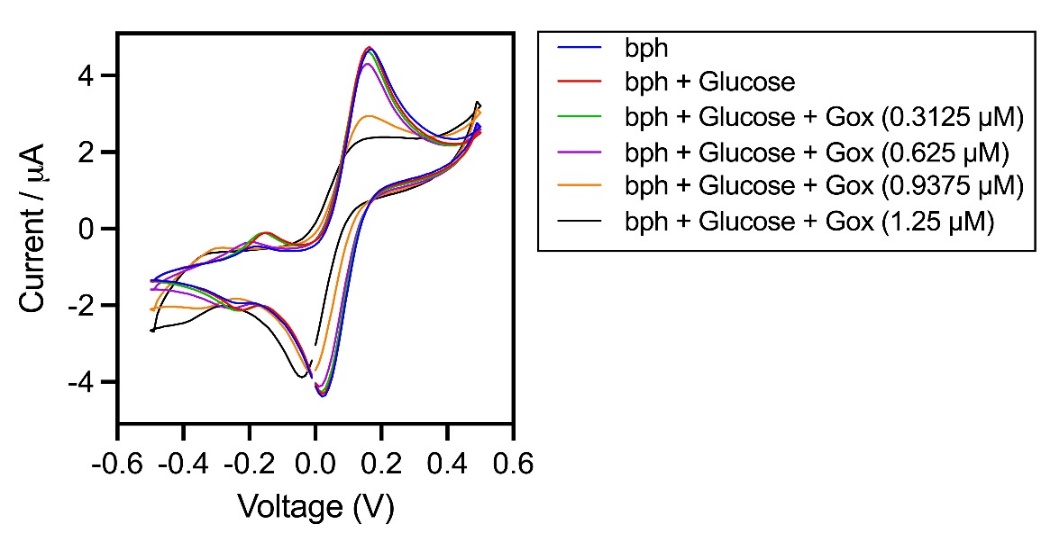

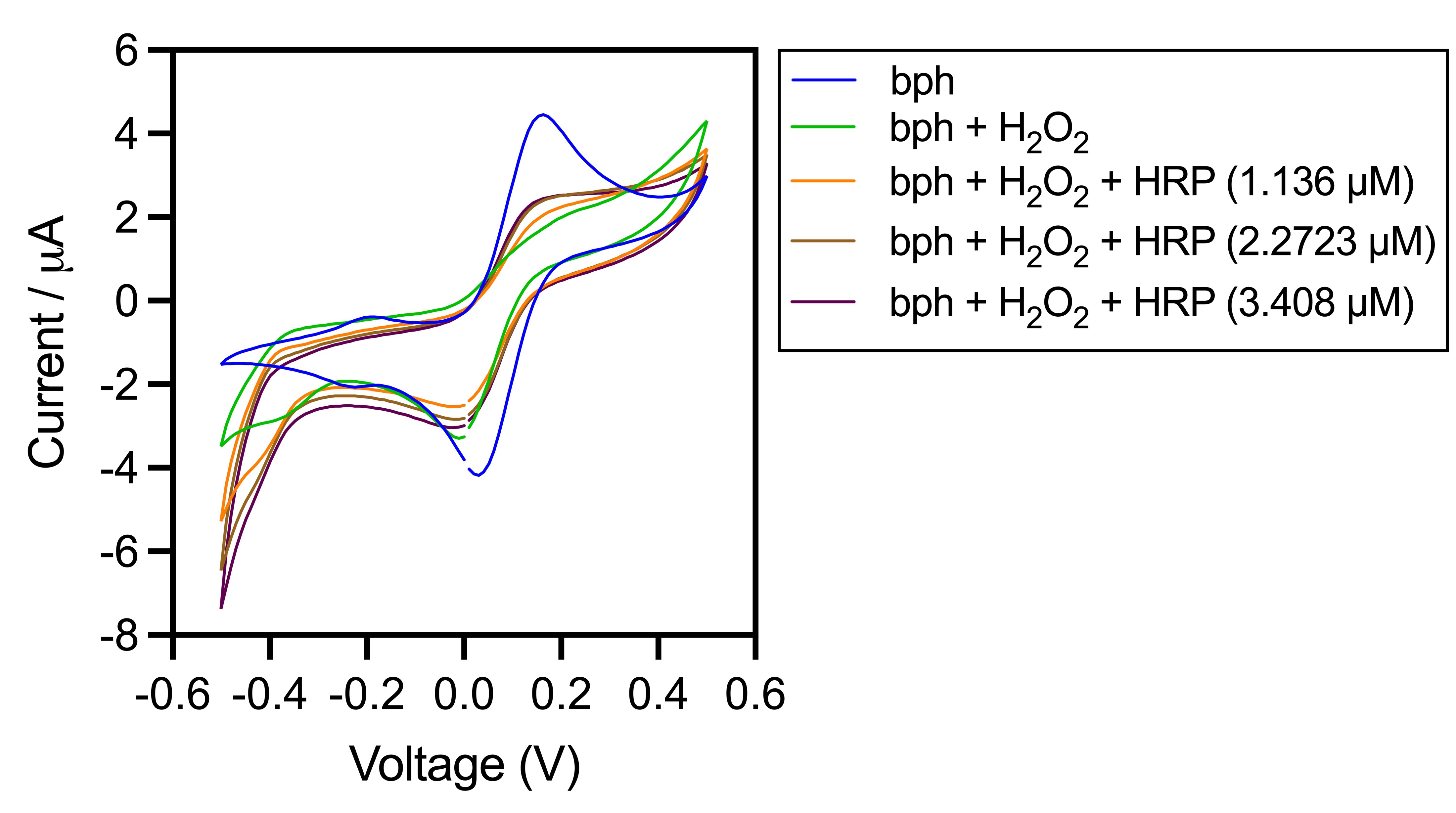


(a)

(b)


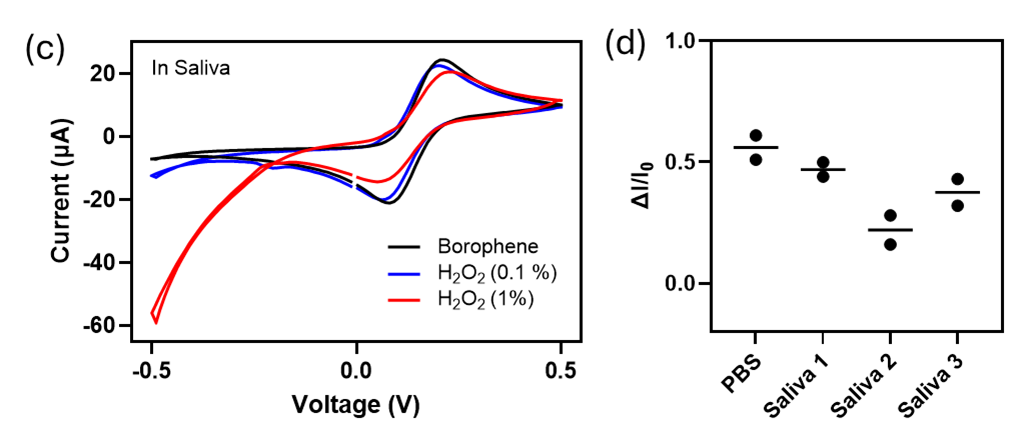


**Figure S3.** Cyclic Voltammetry measurements of a borophene-coated gold screen-printed electrodes with (a) Glucose (0.2 M) with varying concentrations of Glucose Oxidase; (b) H_2_O_2_ (0.2%) with varying concentrations of HRP. (c) Cyclic voltammetry responses of the borophene-modified electrode measured in artificial saliva at different concentrations of hydrogen peroxide exhibited characteristic electrochemical features demonstrating the feasibility of the platform in complex biological environments (d) Change in current output (∆I/I0) was measured in PBS and three saliva samples (n = 3) and results are expressed as mean ± SD.

**
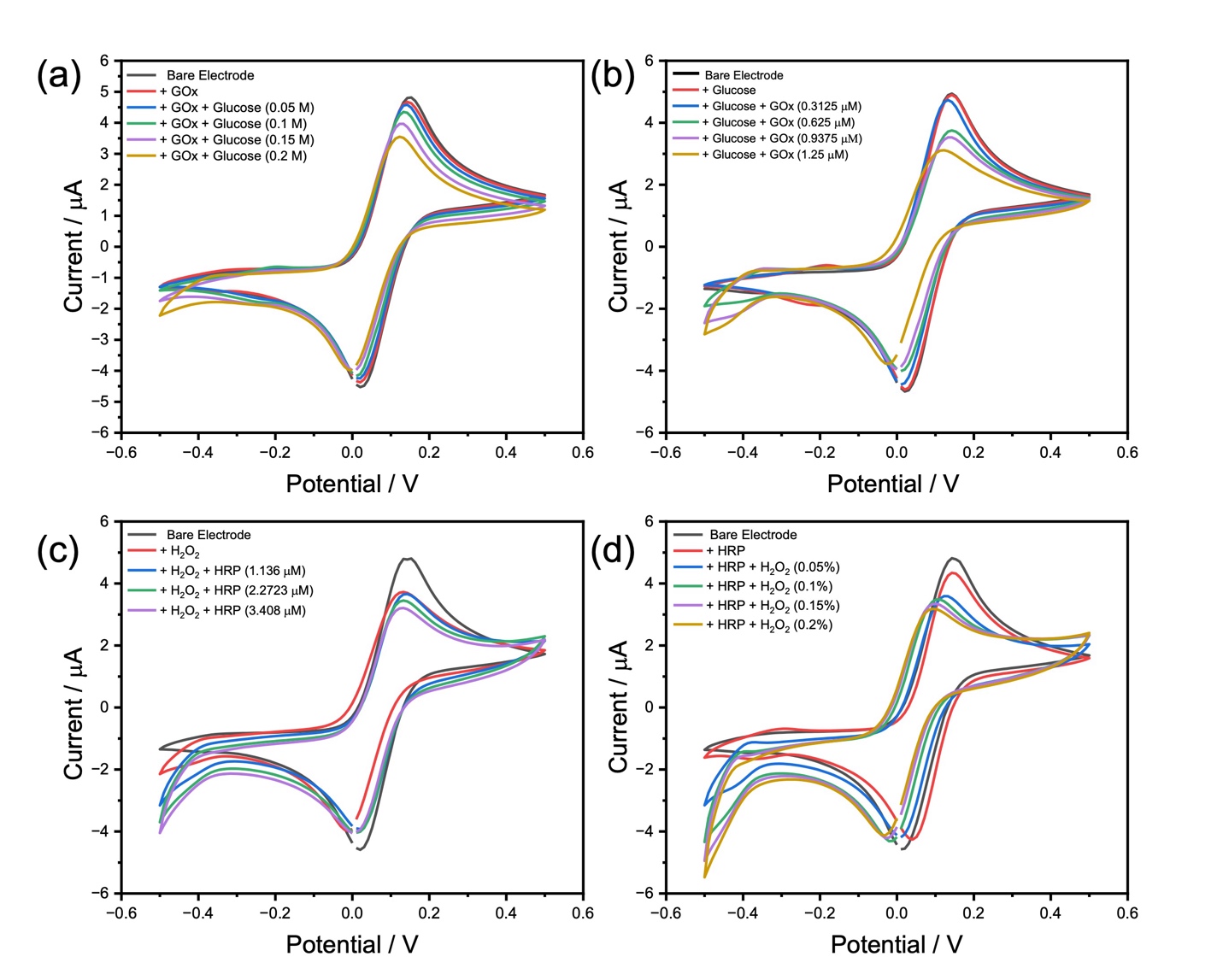
**

**Figure S4.** Cyclic Voltammetry measurements of a bare carbon electrode with (a), (b) Glucose Oxidase and Glucose at varying concentrations; (c), (d) H_2_O_2_ and HRP at varying concentrations


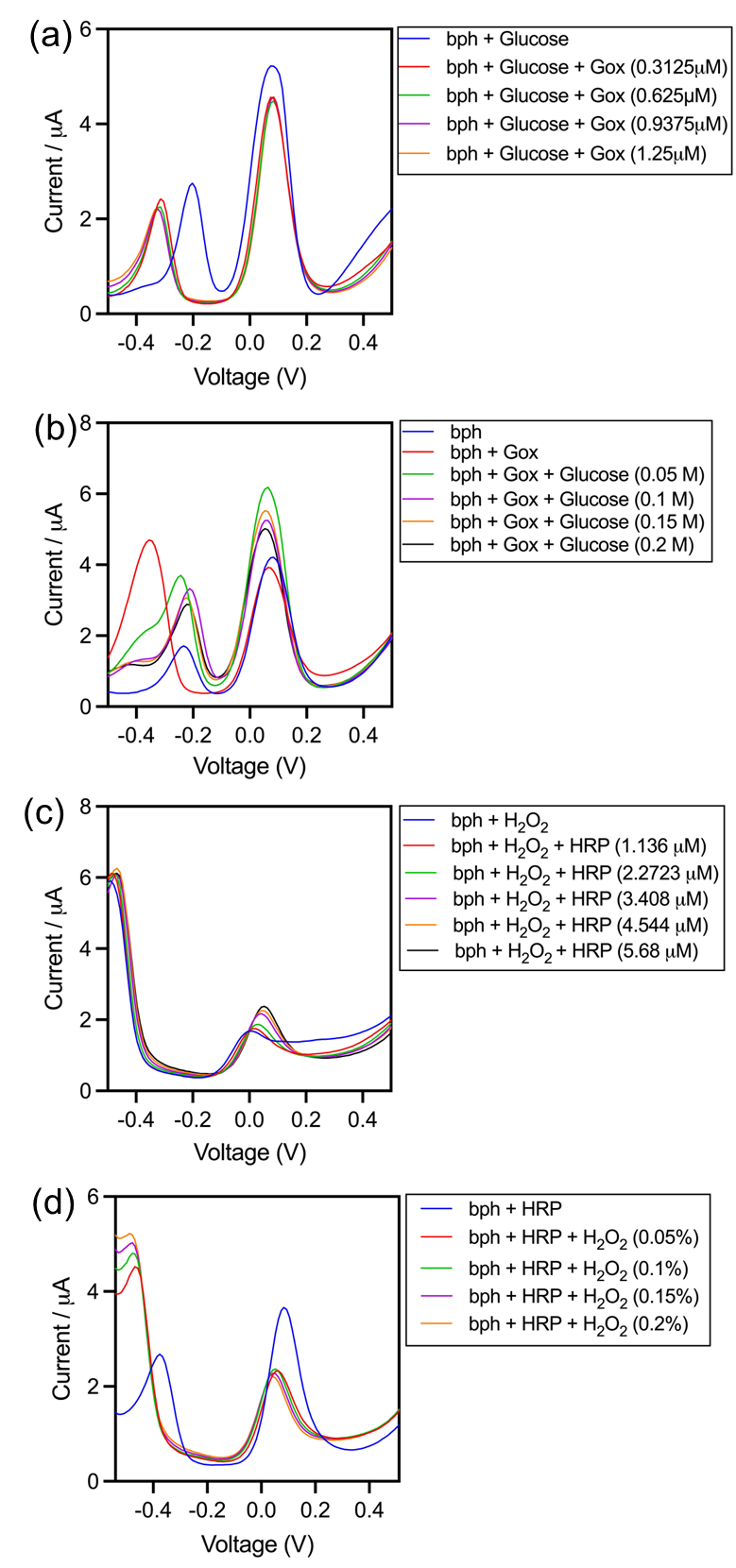


**Figure S5**. Differential Pulse Voltammetry graphs for electrodes coated with borophene (a), (b) Glucose Oxidase and Glucose at varying concentrations; (c), (d) H_2_O_2_ and HRP at varying concentrations.

**Figure S6**. Comparative DPV measurements of graphene, MXene-, and borophene-modified electrodes under identical enzymatic conditions using Glucose oxidase (GOx) at 1.5 uM in the absence and presence of glucose (12.5 mM ) exhibited marked change in current response upon glucose addition, highlighting its superior sensitivity and distinctive ROS-responsive behavior. Change in current output with addition of glucose was also significantly higher for GOx on borophene-modified electrodes (inset, n = 3 and results are expressed as mean ± SD).

**Figure S7.** No significant difference between borophene, graphene, and Mxene-modified electrodes with time, where borophene shows slightly better stability and reproducibility as compared to five electrodes over a period of 45 minutes glucose was also significantly higher for GOx on borophene-modified electrodes (n = 5 and results are expressed as mean ± SD).


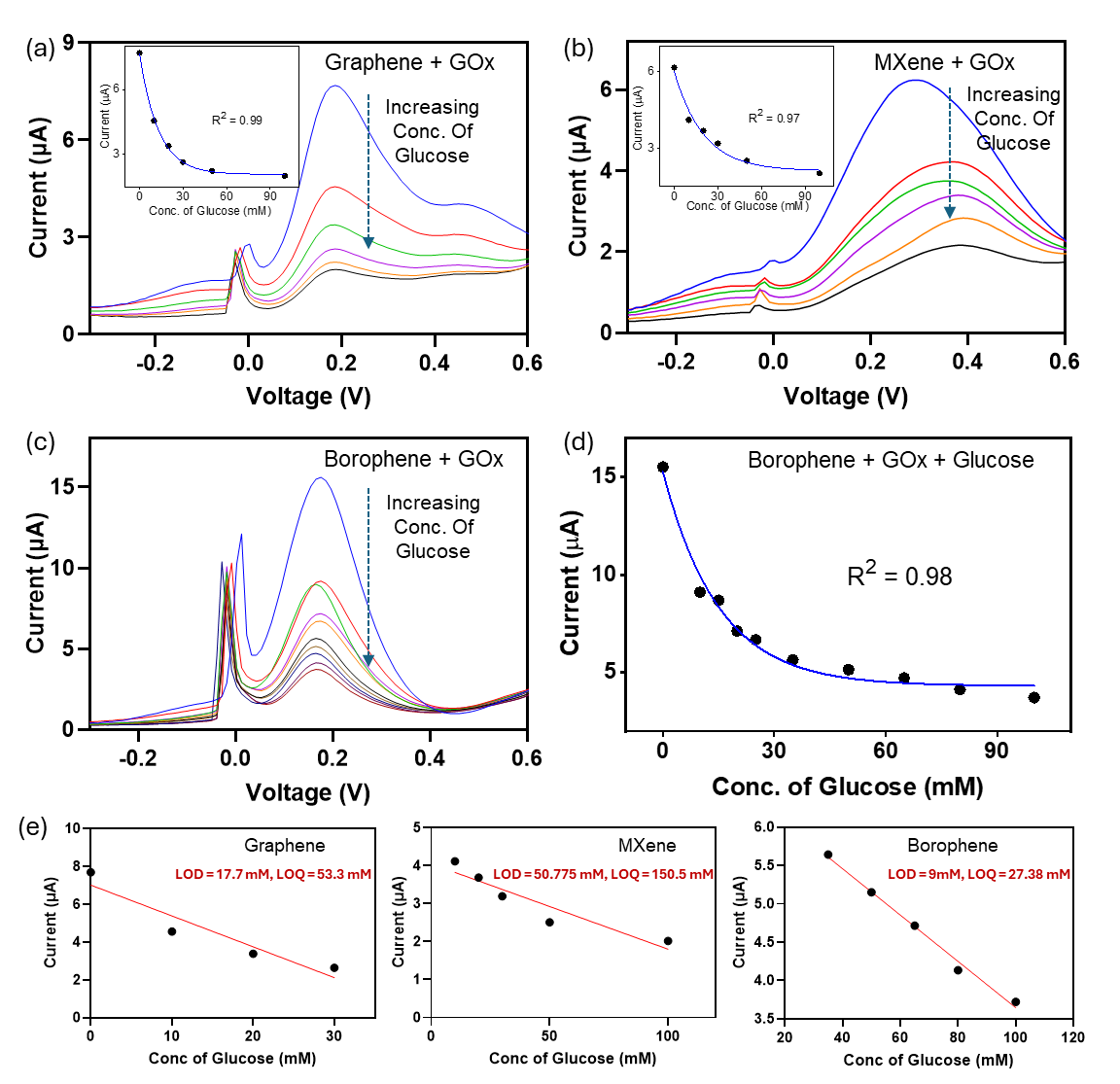


**Figure S8.** Change in differential pulse voltammetry current output upon addition of GOx with glucose for (a) graphene-modified electrodes (LOD = 17.7 mM, LOQ = 53.3 mM), (b) Mxene-modified electrodes (LOD = 50.775 mM, LOQ = 150.5 mM), (c) borophene-modified electrodes (LOD = 9mM, LOQ = 27.38 mM). The nonlinear regression curve fit (in blue) was done and shown in the inset (for graphene and MXene) and in (d) for borophene. (e) The linear regression fit (in red) was done for LOD and LOQ.
